# MicroRNA-497 targets insulin-like growth factor 1 receptor and has a tumour suppressive role in human colorectal cancer

**DOI:** 10.1038/onc.2012.214

**Published:** 2012-06-18

**Authors:** S T Guo, C C Jiang, G P Wang, Y P Li, C Y Wang, X Y Guo, R H Yang, Y Feng, F H Wang, H-Y Tseng, R F Thorne, L Jin, X D Zhang

**Affiliations:** 1 Department of Molecular Biology, Shanxi Cancer Hospital and Institute, Taiyuan, China; 2 Priority Research Centre for Cancer Research, University of Newcastle, Newcastle, New South Wales Australia; 3 Cancer Research Program, Hunter Medical Research Institute, Newcastle, New South Wales Australia; 4 Department of Colorectal Surgery, Shanxi Cancer Hospital and Institute, Taiyuan, China; 5 Cancer Research Unit, School of Biomedical Sciences, University of Newcastle, Newcastle, New South Wales Australia; 6 Immunology and Oncology Laboratory, Kolling Institute of Medical Research, University of Sydney, Sydney, New South Wales Australia

**Keywords:** colorectal cancer, miR-497, IGF1-R, Akt, copy number variations, miRNAs, Colorectal cancer, Cell signalling

## Abstract

Past studies have shown that amplified insulin-like growth factor 1 (IGF1)/IGF1 receptor (IGF1-R) signalling has an important role in colorectal cancer (CRC) development, progression and resistance to treatment. In this report, we demonstrate that downregulation of microRNA-497 (miR-497) as a result of DNA copy number reduction is involved in upregulation of IGF1-R in CRC cells. MiR-497 and miR-195 of the miR-15/16/195/424/497 family that share the same 3′ untranslated region (3′UTR) binding seed sequence and are predicted to target IGF1-R were concurrently downregulated in the majority of CRC tissues relative to paired adjacent normal mucosa. However, only overexpression of miR-497 led to suppression of the IGF1-R 3′UTR activity and downregulation of the endogenous IGF1-R protein in CRC cells. This was associated with inhibition of cell survival, proliferation and invasion, and increased sensitivity to apoptosis induced by various stimuli including the chemotherapeutic drugs cisplatin and 5-fluorouracil, and the death ligand tumour necrosis factor-related apoptosis-inducing ligand. The biological effect of miR-497 on CRC cells was largely mediated by inhibition of phosphatidylinositol 3-kinase/Akt signalling, as overexpression of an active form of Akt reversed its impact on cell survival and proliferation, recapitulating the effect of overexpression of IGF1-R. Downregulation of miR-497 and miR-195 appeared to associate with copy number loss of a segment of chromosome 17p13.1, where these miRs are located at proximity. Similarly to miR-195, the members of the same miR family, miR-424 that was upregulated, and miR-15a, miR-15b and miR-16 that were unaltered in expression in CRC tissues compared with paired adjacent normal mucosa, did not appear to have a role in regulating the expression of IGF1-R. Taken together, these results identify downregulation of miR-497 as an important mechanism of upregulation of IGF1-R in CRC cells that contributes to malignancy of CRC.

**Supplementary information:**

The online version of this article (doi:10.1038/onc.2012.214) contains supplementary material, which is available to authorized users.

## Introduction

Inappropriate activation of survival signalling pathways causes uncontrolled proliferation, resistance to apoptosis and increased motility of cells, and has an important role in cancer development, progression and resistance to treatment.^1, 2^ In colorectal cancer (CRC), one of the mechanisms leading to increased activation of survival signalling pathways, in particular, the phosphatidylinositol 3-kinase (PI3K)/Akt pathway is aberrant expression of type 1 insulin-like growth factor 1 (IGF1) receptor (IGF1-R).^3, 4^ Amplified IGF-1/IGF-1R signalling is not only associated with an increased relative risk for development of CRC, but also contributes to CRC cell survival, invasion, metastasis and resistance to chemotherapeutic drugs.^3, 4, 5, 6, 7, 8^ More recently, it has been shown that IGF1 enriches CRC stem cells, and that targeting IGF1-R results in reduction of CRC stem cell phenotype in colon cancer.^9, 10^

IGF1-R is a transmembrane protein that contains two extracellular α subunits with the ligand-binding site and two transmembrane β subunits with intracellular tyrosine kinase activity.^6, 8^ It is activated by engagement with IGF1 and IGF2 and has an important physiological role in regulating cell growth and proliferation that has been established in knockout mice.^6, 8, 11, 12^ Although upregulation of IGF1-R has been found in many types of cancers including colon cancer,^8^ the mechanism(s) of the increase remains elusive. Recent studies have pointed to a role of microRNAs (miRs) in post-transcriptional regulation of IGF1-R.^13, 14, 15, 16^ While a group of miRs including miR-470, miR-669b and miR-681 are involved in repression of IGF1-R in long-lived mutant mice,^13^ miR-375 and miR-7 target IGF1-R in oesophageal and tongue squamous cell carcinoma cells, respectively.^14, 16^ In addition, miR-145 targets the docking protein of IGF1-R, insulin receptor substrate-1, thus inhibiting IGF1-R signalling.^17^

The expression of miRs is highly tissue and cell type specific, so is the functional significance of an expressed miR.^18, 19, 20^ How these specificities are determined is to be elucidated, but it is known that miRs are frequently regulated by genomic alterations in human cancer.^7, 21^ Like in many other cancer types, the expression and functional importance of miRs in CRC has been studied extensively in recent years.^22, 23, 24, 25, 26, 27^ Although this has resulted in identification of a large number of miRs that are either downregulated or upregulated leading to alterations in the expression of their target genes,^22, 23, 24, 25, 26^ the potential role of miRs in regulating the expression of IGF1-R and its downstream signalling in CRC cells has not been investigated. In this report, we show that miR-497 targets IGF1-R and is frequently downregulated by gene copy number reduction in CRC. In addition, we demonstrate that downregulation of miR-497 contributes to elevated activation of PI3K/Akt signalling and malignant behaviour in CRC cells.

## Results

### MiR-497 is downregulated in CRC cells

**Figure 1:**
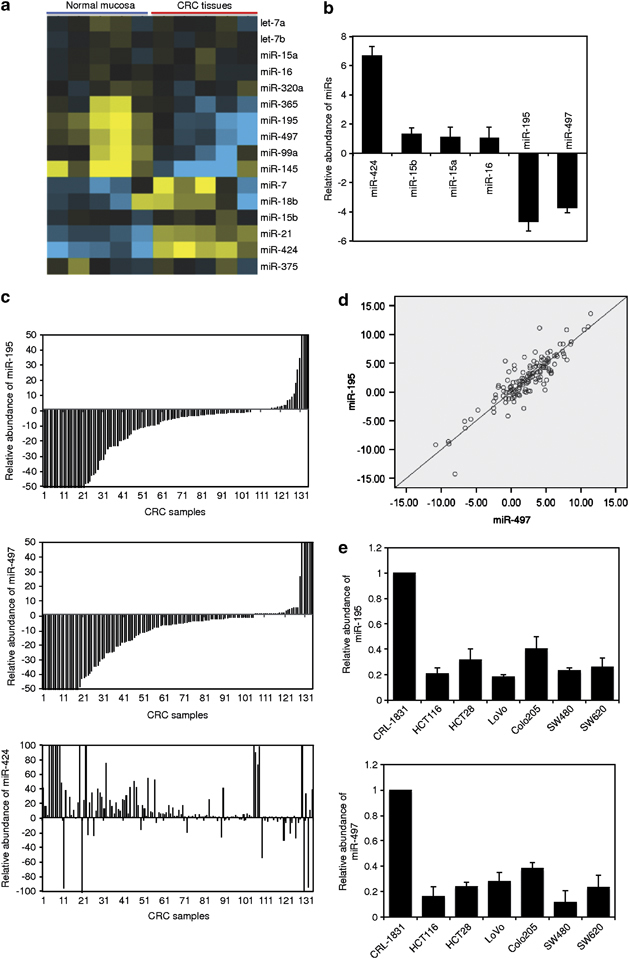
MiR-195 and miR-497 are downregulated in colon cancer cells. (**a**) Total RNA from five representative paired CRC tissues and adjacent normal mucosa were subjected to miR array analysis. The filtered miR array data were subjected to unsupervised hierarchical clustering analysis. The metric was set as the ‘euclidean distance’. (**b**) Quantitative analysis of miR array data showing downregulation of miR-195 and miR-497 and upregulation of miR-424 in CRC tissues compared with paired adjacent normal mucosa. The data shown are average fold changes of individual miR expression in CRC tissues relative to normal mucosa. (**c**) qRT–PCR analysis of miR-195 (upper), miR-497 (middle) and miR-424 (lower) in total RNA from CRC tissues and paired adjacent normal colon mucosa showing that miR-195 and miR-497 are downregulated, whereas miR-424 are upregulated in the majority of colon cancers. The data shown are average fold changes (the mean±s.e. of three individual experiments) of individual miR expression CRC tissues relative to normal mucosa. (**d**) Regression analysis showing that the levels (folds changes in CRC tissues relative to normal mucosa) of miR-195 and miR-497 are significantly correlated in CRC tissues (*R*^2^=0.891, *P*<0.01). (**e**) qRT–PCR analysis of miR-195 (upper) and miR-497 (lower) in total RNA from indicated colon cancer cell lines and the normal colon epithelial line CRL-1831. The data shown are average fold changes (the mean±s.e. of three individual experiments) of individual miR expression in each colon cancer line relative to CRL-1831 cells.

We compared miRNA expression profiles between CRC tissues and paired adjacent non-cancerous mucosa from 10 individual patients using a Human miRNA Microarray Kit (Agilent Technologies, Inc., Santa Clara, CA, USA) that containing 15 024 probes corresponding to 939 human miRNA genes. Among the differentially expressed miRNAs, miR-195 and miR-497 of the miR-15/16/195/424/497 family were expressed respectively more than twofold lower in colon cancer tissues than in normal mucosa. In contrast, miR-424 was markedly upregulated (>2 times) in CRC samples, whereas there was no significant difference (⩽2 times) in the expression levels of miR-15a, miR-15b and miR-16 (Figures 1a and b; Supplementary Table 1). The decreased miR-195 and miR-497 and increased miR-424 expression was confirmed in 137 additional paired samples by qPCR analysis, which showed that miR-195 and miR-497 were downregulated at least twofold in colon cancer tissues in 107 and 106 cases, respectively, and miR-424, upregulated in 99 cases (Figure 1c; Supplementary Table 2). Of note, the levels of miR-195 and miR-497 were significantly correlated (Figure 1d), suggesting that their expression may be regulated in CRC by similar mechanisms.

Despite the alterations in the expression of miR-195, miR-497 and miR-424 in CRC tissues compared with corresponding normal mucosa, there was no significant difference in their expression between pooled early stage (stages I–II) and late stage (stages III–IV) CRC samples, or between those with different anatomic origins (the ascending, the transverse, the descending, the sigmoid colon or the rectum), sex or ages (Supplementary Table 2). These results suggest that downregulation of miR-195 and miR-497 and upregulation of miR-424 may be a common early event in CRC development. Nevertheless, analysis of paired colon adenoma tissues and adjacent normal mucosa (n=8) showed that there was no significant difference in the expression of these miRs (Supplementary Figure 1).

We also examined the expression of miR-195 and miR-497 in a panel of six colon cancer cell lines in comparison with the normal colon epithelial line CRL-1831 by qPCR. Consistent with the results in colon cancer tissues, cultured colon cancer cell lines expressed in general decreased levels of the miRs compared with the CRL-1831 line (Figure 1e). Similarly, miR-424 appeared to be upregulated in colon cancer cell lines, whereas the levels of miR-15a, miR-15b and miR-16 remained unaltered between cultured colon cancer cells and normal colon epithelia cells (Supplementary Figure 2).

### MiR-497 downregulates IGF1-R

**Figure 2:**
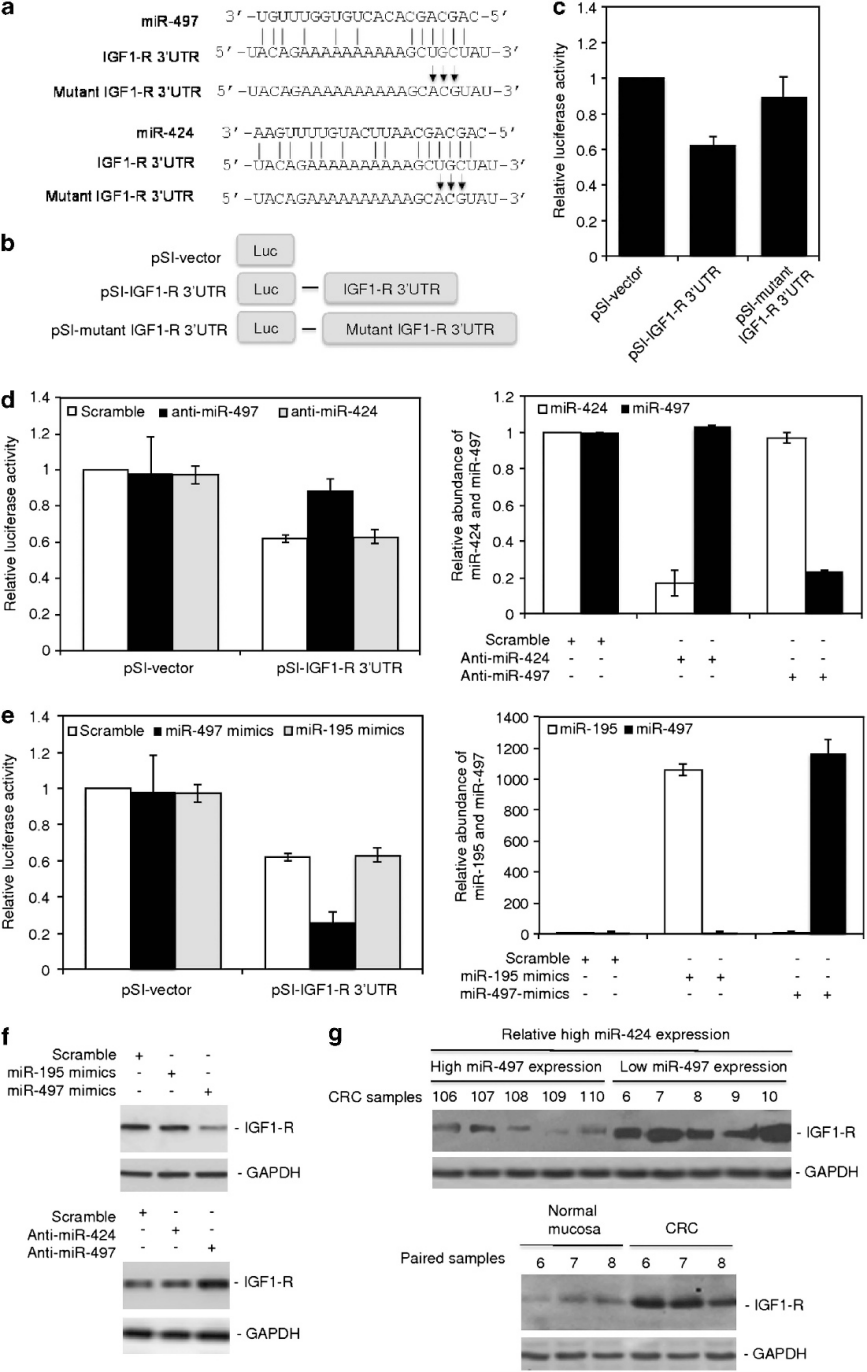
MiR-497 targets IGF1-R. (**a**) A schematic illustration of base paring between miR-497 (upper) and miR-424 (lower) and the 3′UTR of IGF1-R. Substitution of three consecutive bases (UGC to ACG) at the 3′UTR of IGF1-R for the mutant reporter construct is also shown. (**b**) A schematic illustration of the pSI-CHECK2-based luciferase reporter constructs used for examining the effect of miR-424 and miR-497 on the 3′UTR of IGF1-R. (**c**) HCT116 cells were co-transfected with the indicated reporter constructs and *Renilla* luciferase plasmids. Twenty-four hours later, the reporter activity was measured using luciferase assays. The data shown are the mean±s.e. of three individual experiments. (**d**) Left panel: HCT116 cells were co-transfected with the indicated reporter constructs and *Renilla* luciferase plasmids. Scrambled, anti-miR-424 or anti-miR-497 oligonucleotides were also co-transfected. Twenty-four hours later, the reporter activity was measured using luciferase assays. Right Panel: qRT–PCR analysis of miR-424 and miR-497 in total RNA from HCT116 cells transfected with scrambled, anti-miR-424 or anti-miR-497 oligonucleotides. The data shown are the mean±s.e. of three individual experiments. (**e**) Left panel: HCT116 cells were co-transfected with the indicated reporter constructs and *Renilla* luciferase plasmids. Scrambled, miR-195 mimics or miR-497 mimics were also co-transfected. Twenty-four hours later, the reporter activity was measured using luciferase assays. Right Panel: qRT–PCR analysis of miR-195 and miR-497 in total RNA from HCT116 cells transfected with scrambled, miR-195 mimics or miR-497 mimics. The data shown are the mean±s.e. of three individual experiments. (**f**) Upper panel: HCT116 cells were transfected with scrambled, miR-195 mimics or miR-497 mimics. Twenty-four hours later, whole cell lysates were subjected to western blot analysis of IGF1-R and GAPDH (as a loading control). Lower panel: HCT116 cells were transfected with scrambled, anti-miR-424 or anti-miR-497 oligonucleotides. Twenty-four hours later, whole cell lysates were subjected to western blot analysis of IGF1-R and GAPDH (as a loading control). The data shown are representative of three individual western blot analyses. (**g**) Upper panel: western blot analysis of IGF1-R in whole cell lysates from CRC tissues that expressed high levels of miR-424 but different relative (low or high) levels of miR-497 sampled from CRC tissues shown in Figure 1c. Lower panel: western blot analysis of IGF1-R in whole cell lysates from normal colon mucosa. Whole cell lysates from paired colon cancer tissue samples as shown in Figure 1c were included as controls. The data shown are representative of three individual western blot analyses.

One of the predicted common targets of miR-15/16/195/424/497 family members is IGF1-R (http://www.targetscan.org; http://www.ebi.ac.uk/enright-srv/microcosm/htdocs/targets/v5). Since the latter has an important role in colon cancer cell survival, proliferation and resistance to treatment,^5, 6, 7, 28^ we investigated if these miRs impinge on the expression of IGF1-R in colon cancer cells. To this end, we cloned a 1080-bp fragment of the 3′UTR of IGF1-R that contains the binding region of the miRs into a vector with the firefly luciferase reporter gene (Figures 2a and b; Supplementary Figure 3). Introduction of this construct into HCT116 colon cancer cells showed that the reporter activity was suppressed, albeit moderately, by the presence of the 3′UTR of IGF1-R, relative to basal levels observed with the control vector (Figure 2c). This suppression was reversed when the binding region was mutated (Figures 2a–c; Supplementary Figure 3), suggesting that the 3′UTR of IGF1-R was inhibited by the endogenous expression of one or more members of this miR family, in particular, miR-424 that is increased in CRC cells. However, inhibition of miR-424 by co-introduction of anti-miR-424 did not alter the report activity (Figure 2d). Similarly, co-introduction of anti-miR-15a, anti-miR-15b, anti-miR-16 or anti-miR-195 did not cause any significant change in the activity (Supplementary Figure 4). In contrast, co-introduction of anti-miR-497 triggered an increase in the report activity (Figure 2d), indicating that miR-497, but not the other miRs, has a role in inhibiting the 3′UTR of IGF1-R. In support, the addition of miR-497 mimics, but not the addition of miR-195, miR-15a, miR-15b, miR-16 or miR424 mimics, further inhibited the reporter activity (Figure 2e; Supplementary Figure 5). Consistent with the results of luciferase assays, introduction of miR-497 mimics downregulated, whereas introduction of anti-miR-497 upregulated endogenous IGF1-R protein levels. On the other hand, introduction of miR-195 mimics or anti-miR-424 had no effect on the expression of IGF1-R (Figure 2f).

The effect of miR-497 on the expression of IGF1-R in colon cancer cells was further consolidated by examination of representative colon cancer tissues that expressed increased levels of miR-424 (>2 times) compared with normal mucosa and were sampled by relatively low (*n*=5) and high (*n*=5) levels of miR-497 (Figure 1c; Supplementary Figure 6). Regardless of the high levels of miR-424, colon cancers with low miR-497 expression displayed relatively high levels of IGF1-R, whereas high miR-497 expression was associated with relatively low levels of IGF1-R (Figure 2g). Similarly, normal colon cancer mucosa that had in general higher levels of miR-497 than colon cancer tissues expressed relatively low levels of IGF1-R (Figure 2g; Supplementary Figure 6).

### MiR-497 inhibits PI3K/Akt signalling

**Figure 3:**
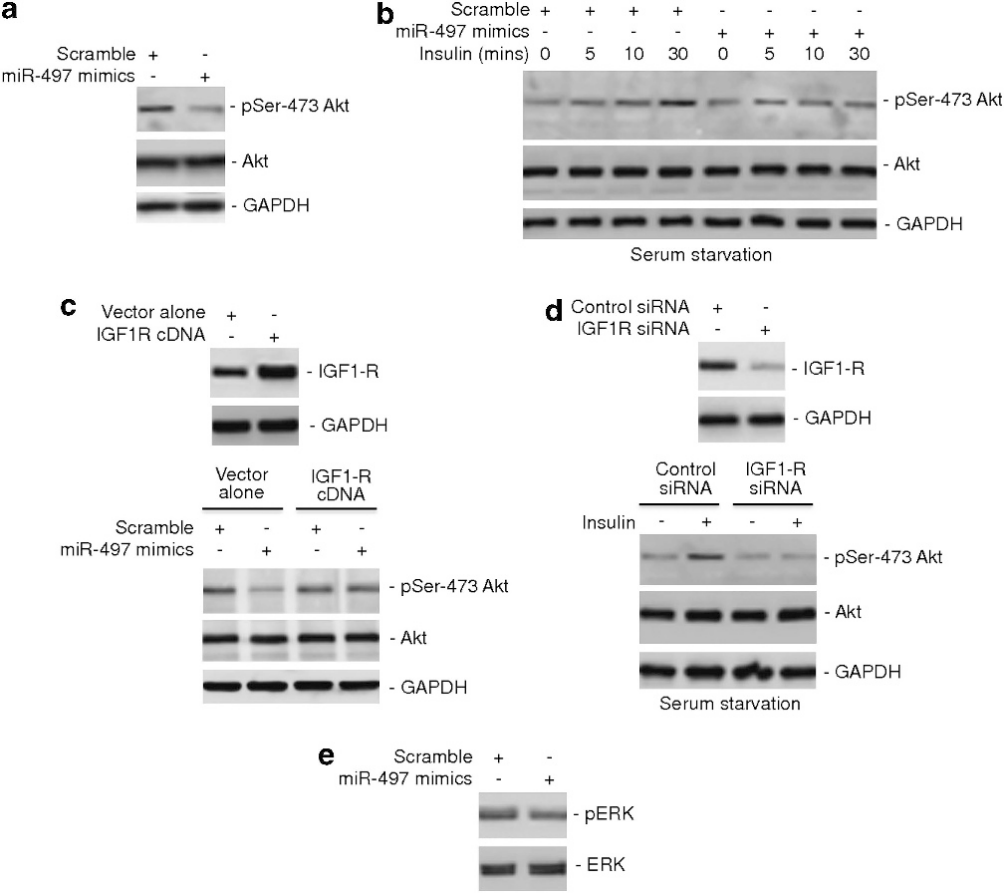
MiR-497 inhibits activation of Akt. (**a**) Whole cell lysates from HCT116 cells transfected with scrambled or miR-497 mimics were subjected to western blot analysis of phosphorylated Akt (pSer-473-Akt), total Akt (Akt) and GAPDH (as a loading control). The data shown are representative of three individual western blot analyses. (**b**) HCT116 cells transfected with scrambled or miR-497 mimics were subjected to serum starvation for 6 h before the addition of recombinant insulin (1 μM) for indicated periods. Whole cell lysates were subjected to western blot analysis of pSer-473-Akt, Akt and GAPDH (as a loading control). The data shown are representative of three individual experiments. (**c**) Upper panel: whole cell lysates from HCT116 cells co-transfected with miR-497 mimics and vector alone or IGF1-R cDNA were subjected to western blot analysis of IGF1-R and GAPDH (as a loading control). Lower panel: whole cell lysates from HCT116 cells co-transfected with scrambled or miR-497 mimics and vector alone or IGF1-R cDNA were subjected to western blot analysis of pSer-473-Akt, Akt and GAPDH (as a loading control). The data shown are representative of three individual experiments. (**d**) Upper panel: whole cell lysates from HCT116 cells transfected the control or IGF1-R siRNA were subjected to western blot analysis of IGF1-R and GAPDH (as a loading control). Lower panel: HCT116 cells were transfected with the control or IGF1-R siRNA. Twenty-four hours later, cells were treated with recombinant insulin (1 μM) for 30 min. Whole cell lysates were then subjected to western blot analysis of pSer-473-Akt, Akt and GAPDH (as a loading control). The data shown are representative of three individual experiments. (**e**) Whole cell lysates from HCT116 cells transfected with scrambled or miR-497 mimics were subjected to western blot analysis of phosphorylated ERK (pERK) and ERK. The data shown are representative of three individual western blot analyses.

Since IGF1-R has an important role in mediating activation of the PI3K/Akt pathway,^3, 28^ we focused on examining whether miR-497-triggered suppression of IGF1-R is reflected in regulation of PI3K/Akt signalling in colon cancer cells. As expected, introduction of miR-497 mimics into HCT116 cells caused inhibition of constitutive Akt activation as determined by the expression of phosphorylated Akt (pSer473-Akt) (Figure 3a). Consistently, miR-497 mimics inhibited activation of Akt in response to stimulation with insulin in HCT116 cells under serum starvation conditions (Figure 3b). Of note, the levels of Akt activation in cells introduced with miR-497 mimics appeared similar to those in cells introduced with scramble controls after serum starvation, reiterating that the inhibitory effect of miR-497 on activation of Akt is mediated by blockage of upstream signalling resulting from extracellular stimulation, consistent with its role in inhibition of IGF1-R. In support, overexpression of IGF1-R abolished the inhibitory effect of miR-497 mimics on constitutive activation of Akt (Figure 3c), whereas knockdown of IGF1-R recapitulated the effect miR-497 on Akt activation in response to insulin stimulation under serum starvation conditions (Figure 3d). These results indicate that miR-497 has an important role in regulation of activation of PI3K/Akt signalling in colon cancer cells. Similarly to activation of Akt, activation of ERK was also downregulated, albeit moderately, when miR-497 was overexpressed, suggesting that miR-497 may also have a role in regulating activation of the MEK/ERK signalling pathway (Figure 3e).

### MiR-497 inhibits proliferation and invasive behaviour in colon cancer cells

**Figure 4:**
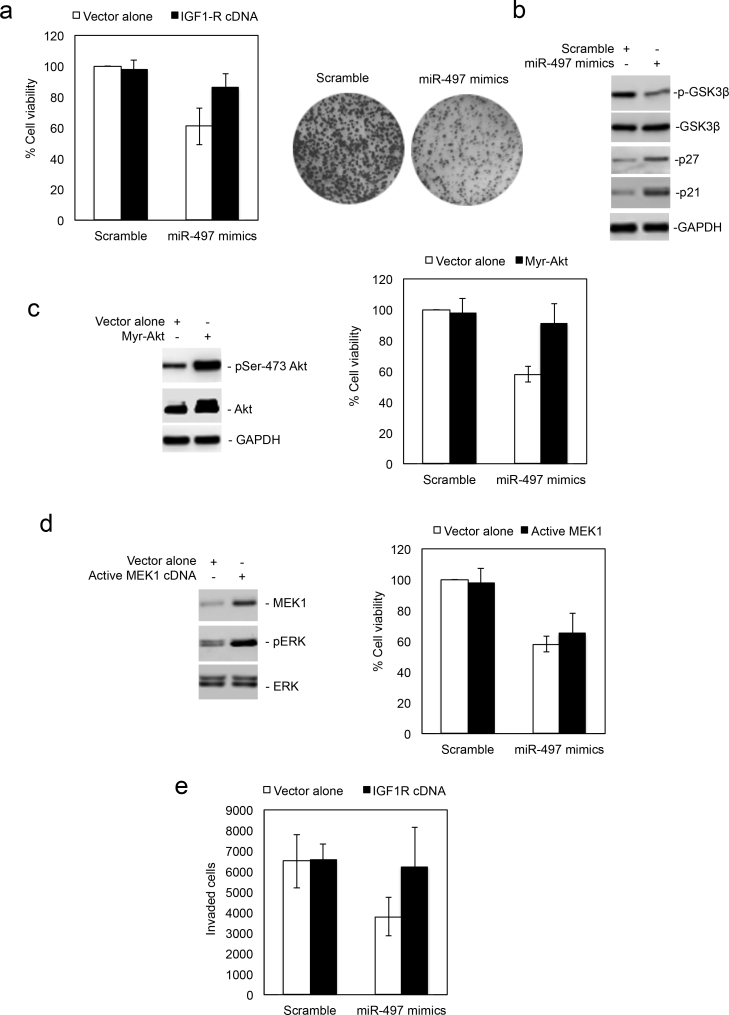
MiR-497 inhibits proliferation and invasive potential of colon cancer cells. (**a**) Left panel: HCT116 cells were co-transfected with scrambles or miR-497 mimics and vector alone or IGF1-R cDNA were subjected to analysis of cell viability by MTS assays. The data shown are the mean±s.e. of three individual experiments. Right panel: HCT116 cells were transfected with scrambled or miR-497 mimics. Twenty-four hours later, viable cells were harvested and reseeded at 2000 cells/well onto 6-well plates as single cell suspension. Cells were allowed to grow for 12 days before being fixed with methanol and stained with crystal violet. The data shown are representative of three individual experiments. (**b**) Whole cell lysates from HCT116 cells transfected with scrambled or miR-497 mimics were subjected to western blot analysis of phosphorylated GSK3β (p-GSK3β), total GSK3β,p27, p21 and GAPDH (as a loading control). The data shown are representative of three individual experiments. (**c**) Left panel: HCT116 cells were co-transfected with miR-497 mimics and vector alone or an myr-Akt construct. Twenty-four hours later, whole cell lysates were subjected to western blot analysis of pSer-473 Akt, Akt and GAPDH (as a loading control). The data shown are representative of three individual experiments. Right panel: HCT116 cells co-transfected with scrambled or miR-497 mimics and vector alone or an myr-Akt construct were subjected to analysis of cell viability by MTS assays. The data shown are mean±s.e.m. of three individual experiments. (**d**) Left panel: HCT116 cells were co-transfected with miR-497 mimics and vector alone or a constitutive active MEK1 construct. Twenty-four hours later, whole cell lysates were subjected to western blot analysis of MEK1, phosphorylated ERK (pERK) and ERK. The data shown are representative of three individual experiments. Right panel: HCT116 cells co-transfected with scrambled or miR-497 mimics and vector alone or a constitutive active MEK1 construct were subjected to analysis of cell viability by MTS assays. The data shown are mean±s.e.m. of three individual experiments. (**e**) HCT116 cells were co-transfected with scrambled or miR-497 mimics and vector alone or IGF1-R cDNA. Twenty-four hours later, viable cells were harvested and reseeded onto transwell chambers containing Matrigel as a barrier in medium containing 10% fetal calf serum. Forty-eight hours after seeding, invaded cells were detached, stained and quantitated. The data shown are mean±s.e.m. of three individual experiments.

Having demonstrated that miR-497 regulates activation of the PI3K/Akt pathway, we examined whether it has a role in regulation of colon cancer cell survival and proliferation. While introduction of miR-497 mimics did not cause significant apoptosis in HCT116 cells (around 10% apoptotic cells; Figure 2e and Supplementary Figure 7), it did result in more significant inhibition of cell viability and proliferation (Figure 4a). Associated with this was the decreased phosphorylation of GSK3β and increased expression of p27 and p21, downstream targets of Akt signalling that are important in regulating cell-cycle progression (Figure 4b). Co-introduction of an IGF1-R-expressing construct, or an activated form of Akt (myr-Akt), to HCT116 cells, partially blocked inhibition of cell viability by miR-497 mimics^29^ (Figures 4a and c), indicating that reduced cell viability in colon cancer cells when miR-497 is overexpressed is related to downregulation of IGF1-R and decreased activation of Akt signalling. In contrast, overexpression of an active form of MEK1 had only a minimal effect on inhibition of cell viability mediated by miR-497 mimics (Figure 4d), suggesting that the moderate inhibition of the MEK/ERK pathway does not have a major role in reduced cell viability when miR-497 is overexpressed in colon cancer cells.

Because the PI3K/Akt pathway also has an important role in regulating migration and invasion of malignant cells,^1, 2^ we investigated whether the regulatory effect of miR-497 on IGF1-R/PI3K/Akt signalling is also reflected in its impact on colon cancer cell invasive behaviour. To this end, we seeded HCT116 cells that had been introduced with the scramble sequences or miR-497 mimics for 24 h onto Boyden chambers coated with Matrigel. Cells treated with miR-497 mimics displayed reduced ability to invade through Matrigel in comparison with those treated with scrambled sequences, which was efficiently reversed by overexpression of IGF1-R (Figures 2e, 3c, and 4e). These results suggest that that miR-497-mediated downregulation of IGF1-R also has a role in inhibition of invasive behaviour of colon cancer cells.

### MiR-497 confers increased sensitivity of colon cancer cells to apoptosis

**Figure 5:**
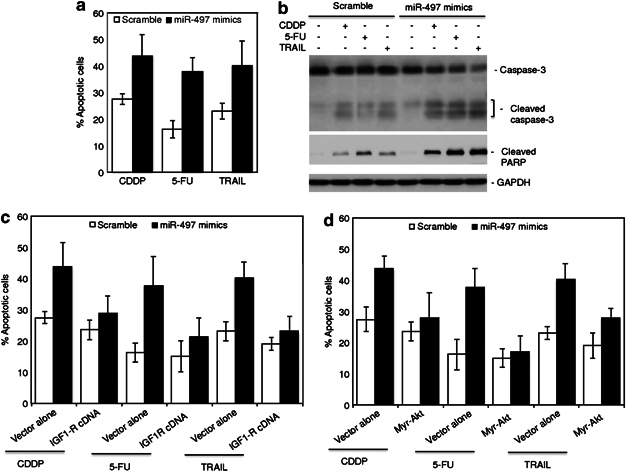
MiR-497 sensitizes colon cancer cells to apoptosis. (**a**) HCT116 cells were transfected with scrambled or miR-497 mimics. Twenty-four hours later, cells were treated with CDDP (5 μg/ml) or 5-FU (5 mg/ml) for a further 48 h, or TRAIL (100 ng/ml) for a further 24 h. Apoptosis was quantitated by measurement of sub-G1 DNA content. The data shown are mean±s.e.m. of three individual experiments. (**b**) HCT116 cells were transfected with scrambled or miR-497 mimics. Twenty-four hours later, cells were treated with CDDP (5 μg/ml) or 5-FU (5 mg/ml) for a further 24 h, or TRAIL (100 ng/ml) for a further 12 h. Whole cell lysates were subjected to western blot analysis of caspase-3, cleaved PARP and GAPDH (as a loading control). The data shown are representative of three individual experiments. (**c**) HCT116 cells were co-transfected with scrambled or miR-497 mimics and vector alone or IGF1-R cDNA. Twenty-four hours later, cells were treated with CDDP (5 μg/ml) or 5-FU (5 mg/ml) for a further 48 h, or TRAIL (100 ng/ml) for a further 24 h. Apoptosis was quantitated by measurement of sub-G1 DNA content. The data shown are mean±s.e.m. of three individual experiments. (**d**) HCT116 cells were co-transfected with scrambled or miR-497 mimics and vector alone or an myr-Akt construct. Twenty-four hours later, cells were treated with CDDP (5 μg/ml) or 5-FU (5 mg/ml) for a further 48 h, or TRAIL (100 ng/ml) for a further 24 h. Apoptosis was quantitated by measurement of sub-G1 DNA content. The data shown are mean±s.e.m. of three individual experiments.

Although overexpression of miR-497 did not cause significant apoptosis (Supplementary Figure 7), it remains possible that miR-497 impinges on sensitivity of colon cancer cells to apoptosis induced by other stimuli. To test this, HCT116 cells introduced with scramble controls or miR-497 mimics were exposed to the DNA-damaging drug, cisplatin, the thymidylate synthase inhibitor, 5-FU, and the death ligand tumour necrosis factor-related apoptosis-inducing ligand (TRAIL). Overexpression of miR-497 resulted in increased sensitivity of the cells to apoptosis induced by all these apoptotic stimuli, as shown by accumulation of sub-G1 DNA content, enhanced activation of caspase-3 and cleavage of its substrate PARP (Figures 5a and b). This sensitization was, at least in part, due to downregulation of IGF1-R and inhibition of Akt activation, in that co-introduction of an IGF1-R-expressing construct blocked enhancement in induction of apoptosis afforded by overexpression of miR-497 mimics (Figures 3c and 5c), and that co-introduction of an activated form of Akt (myr-Akt) recapitulated the inhibitory effect of IGF1-R (Figures 4c and 5d). Similarly, the general caspase inhibitor z-VAD-fmk or co-introduction of a Bcl-2-expressing construct inhibited induction of apoptosis even when miR-497 was overexpressed (Supplementary Figure 8), pointing to the involvement of the caspase cascade and the mitochondrial apoptotic pathway.

### Downregulation of miR-497 is associated with DNA copy number reduction

**Figure 6:**
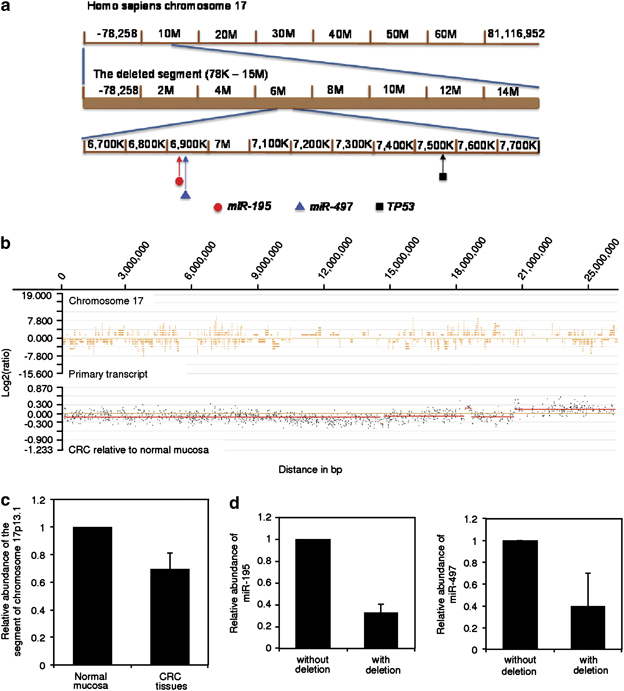
Downregulation of miR-195 and miR-497 in colon cancer cells is associated with DNA copy number reduction. (**a**) A schematic illustration of deletion of a segment (78K–15M) of chromosome 17p13.1 where the *miR-195* and *miR-497* genes are located. (**b**) Representative aCGH analysis results showing copy number loss of a segment (78K–15M) of chromosome 17p13.1 in CRC tissues relative to normal colon mucosa. (**c**) Genomic qPCR analysis of total RNA from 131 paired CRC tissues and normal mucosa confirming that the copy number of a fragment (78K–15M) of chromosome 17p13.1 is frequently reduced in CRC tissues. The average copy number of the fragment in normal mucosa was arbitrarily designated as 1. The data shown are the mean±s.e. of results from 131 CRC tissues. (**d**) qPCR analysis of total RNA from 131 paired CRC tissues showing that miR-195 (left) and miR-497 (right) are expressed at reduced levels in those with copy number reduction in the fragment (78K–15M) of chromosome 17p13.1. The level of miR-195 or miR-497 in CRC tissues without copy number reduction in the fragment was arbitrarily designated as 1. The data shown are the mean±s.e. of results from 131 CRC tissues in three individual experiments.

The finding that miR-497 and its relative miR-195 are concurrently downregulated in the majority of colon cancer tissues suggests that these miRNAs may be regulated by similar mechanisms. One possibility is that their downregulation is due to genomic alterations that are frequently involved in regulation of miRNA expression.^7, 21^ Indeed, the *miR-497* and *miR-195* genes are located at proximity to a segment of chromosome 17p13.1 (Figure 6a), which was found to be deleted in 6 of 10 colon cancer samples compared with corresponding normal mucosa by array comparative genomic hybridization (aCGH; Figures 6a and b). Copy number reduction at this fragment (78K–15M) was confirmed in the cohort of 131 paired CRC tissue and normal mucosa samples by genomic qPCR, which showed that ∼71% of colon cancers had DNA copy number reduction at this segment (Figure 6c). Of note, the levels of miR-497 and miR-195 were significantly lower in colon cancer samples with deletion of the segment of chromosome 17p13.1, indicating that downregulation of these miRNAs in colon cancers is closely related to their DNA copy number reduction (Figure 6d). As a control, the product of the tumour suppressor gene *TP53* that maps to the same fragment of chromosome 17p13.1 was also markedly lower in colon cancer samples with deletion of the locus (Supplementary Figure 9).

## Discussion

In this report, we present evidence that miR-497 has an important role in inhibiting IGF1-R expression and activation of PI3K/Akt signalling, and in suppressing proliferation, survival and invasive behaviour in human colon cancer cells. While its expression is commonly reduced in colon cancer cells *in vitro* and *in vivo* relative to normal colon epithelial tissues, introduction of exogenous miR-497 into colon cancer cells resulted in downregulation of IGF1-R and decreased activation of Akt. This was associated with inhibition of cell proliferation, decreased invasive potential and enhancement in sensitivity to apoptosis. Our results also associate the downregulation of miR-497 in colon cancer cells with the genomic deletion of a fragment of chromosome 17p13.1, where miR-497 maps.

MiR-497 belongs to the miR-15/16/195/424/497 family. Members of this miR family share the same 3′UTR binding seed sequence and have been reported to cooperatively regulate a number of targets, including CDK6, CARD10 and CDC27 leading to regulation of cell proliferation in concert in colon cancer cells.^30^ Similarly, miR-15, -16, -195 and -497 have been shown to target Bcl-2, thus promoting induction of apoptosis, although these findings were made in different cell types.^27, 31, 32, 33, 34^ However, unlike miR-497, miR-15, -16, -195 and -424 did not appear to target the 3′UTR of IGF1-R in colon cells. The reason for this discrepancy remains to be defined, but it is known that seed pairing may not invariably be a reliable predictor for miR-target interaction.^35, 36^ Other factors, such as AU-rich nucleotide composition and relative position of binding sites to the stop codon may also have a part in determining miR targeting specificity.^35, 36^ It is well documented that while functional consequences of miR expression are commonly tissue and cell type specific, regulation of target expression by miRs is also in a cell type-dependent manner.^7, 20^ Of note, results from this study and others indicated that there were wide variations in the expression of many miRs among normal colon mucosa samples,^37^ suggesting that the expression of miRs in normal tissues is also highly individual dependent.^37, 38, 39^ Whether this is related to patients’ age, gender, nutritional conditions and other factors requires further investigation. The miR expression viability in human cervical tissues has been suggested to be associated with the presence or absence of the human papillomavirus in samples.^39^

The differential expression of a number of miRs in colon cancer tissues has been proposed to be potential biomarkers for prognosis and responses to treatment.^22, 23, 24, 25, 26, 27^ For example, the increased expression of miR-21 was identified as an independent predictor of overall survival of colon cancer patients, whereas let-7g and miR-18b expression was closely associated with resistance of colon cancer to treatment with S-1, an analogue of 5-FU.^40, 41^ In addition, downregulation of miR-195 has been shown to correlate with poor prognosis of patients with colon cancer.^42^ However, because most patients from whom the colon cancer and control tissues were obtained and analysed in this study are still alive, we are unable to conclude at present whether the reduced expression of miR-497 is of significance in predicting disease progression and prognosis of patients. Nevertheless, our results indicate that the levels of miR-497 are correlated with the levels of miR-195 in colon cancer tissues, suggesting that downregulation of miR-497 may, similarly to the reduced expression of miR-195, associate with poor prognosis of colon cancer patients.^42^ Moreover, our finding showing that downregulation of miR-497 and miR-195 and upregulation of miR-424 may be early events in colon cancer development suggest that the altered expression of these miRNAs may be a useful biomarker in diagnosis of colon cancer.

MiR-encoding genes are frequently located to clusters at fragile sites in the genome where genomic alterations frequently take place.^7, 21^ In colon cancer, particular chromosomal regions are commonly gained and overexpressed or lost and underexpressed in comparison with normal colon mucosa, suggesting that these changes may be tumorigenic.^43^ In this study, we found that a segment of chromosome 17p13.1 where both miR-497 and miR-195 are located was frequently deleted in colon cancer cells, indicating that downregulation of these miRs in the cells is related to gene copy number reduction. This was further supported by the result showing that colon cancer samples with deletion of the fragment had lower levels of miR-497 and miR-195 than those without the deletion. Consistently, downregulation of miR-497 and miR-195 as a consequence of copy number reduction of chromosome 17p13.1 has been reported in peritoneal carcinoma cells.^44^ Of note, the tumour suppressor gene *TP53* also maps to the same fragment of chromosome 17p13.1, which is frequently underexprssed in colon cancer cells.^45^ Together, these results suggest that deletion of the segment of chromosome 17p13.1 may have a role in colon cancer tumorigenesis, whereas miR-497 and miR-195, like p53, may have potential roles as tumour suppressors. In support of our results, deletions of parts of chromosomal 17p have been previously reported in colon cancer cells.^43^

The functional significance of miR-497 in regulating malignant behaviour of colon cancer cells is echoed by its downregulation in colon cancer tissues *in vivo*, and is further supported by the negative association between its expression levels and the levels of IGF1-R. The increased expression of the latter has an important role in colon cancer cell proliferation, invasion and resistance to apoptosis by activation of survival signalling pathways, in particular, the PI3K/Akt pathway.^3, 4, 5, 6, 7^ Targeting IGF1-R is in development for clinical use in the treatment of cancer.^46, 47^ Our results suggest that restoration of the expression of miR-497 may be a useful alternative strategy for inhibition of IGF1-R in CRC. As a precedent, replacement therapy has been shown to be efficacious in a mouse model of colon cancer for miR-145 and miR-33a.^48^ With continuing investigations into *in-vivo* delivery systems for miRs this approach is likely to be achievable.

## Materials and methods

### Cell culture and reagents

Human colon cancer cell lines HCT116, HCT28, LoVo, Colon205, SW480 and SW620 were obtained from the American Type Culture Collection (ATCC, Rockville, MD, USA) and Dr Shafren DR (Faculty of Health, University of Newcastle, NSW, Australia), and were maintained as monolayers in DMEM containing 5% FCS (Commonwealth Serum Laboratories, Melbourne, VIC, Australia). The normal fetal human colon epithelia cell line CRL-1831 was from ATCC and Dr Chen JZ (Illawarra Health and Medical Research Institute, University of Wollongong, Australia), and were cultured in a 1:1 mixture of Ham’s F12 and modified DMEM containing HEPES (25 mM), cholera toxin (10 ng/ml), insulin (5 μg/ml), transferrin (5 μg/ml) and hydrocortisone (100 ng/ml) (Sigma-Aldrich, Castle Hill, NSW, Australia). Recombinant human insulin was purchased from Sigma-Aldrich. Antibodies against Akt, phospho-Akt (Ser473), ERK, MEK1, phosphorylated GSK3β and GSK3β, and IGF1-R were from Cell Signalling Technology (Beverly, MA, USA). Antibody against p-ERK and Bcl-2 were purchased from Santa Cruz Biotechnology (Santa Cruz, CA, USA). Antibodies against p27, PARP and p53 were purchased from BD Biosciences (Bioclone, Marrickville, NSW, Australia). The antibody against Caspase-3 was from Stressgen (Victoria, BC, Canada). The cell-permeable general caspase inhibitor Z-Val-Ala-Asp(OMe)-CH2F (z-VAD-fmk) was purchased from Calbiochem (Merck KGaA, Darmstadt, Germany). Mature has-miR-195, -497, -15a, -15b, -16 or -424 mimics (double-stranded oligonucleotides designed to mimic the function of endogenous miR) and anti-has-miR-195, -497, -15a, -15b, -16 or -424 (antisense RNA oligonucleotides designed to inhibit the function of endogenous miR) were purchased from Thermo Fisher Scientific (North Ryde, NSW, Australia).

### Human tissue samples

Human CRC samples and paired normal mucosa were obtained from patients undergoing surgical resection of sporadic CRCs in Shanxi Cancer Hospital. The study was approved by Human Research Ethics Committee of the hospital. All participants provided written informed consent. In every case, a histopathological diagnosis of CRC had been ascertained, and a family history of CRC had been excluded. All tissue samples were selected by an experienced pathologist immediately after surgical resection, snap frozen in liquid nitrogen, and then stored at −80 °C. Age, gender and staging information is given in Supplementary Table 2.

### miR microarray

RNA labelling and hybridization on miRNA microarray chips were performed as previously described^49, 50^ by CapitalBio (Capital-Bio Corp., Beijing, China). Briefly, 100 μg total RNA was purified by using mirVANA miRNA isolation kit (Ambion, Austin, TX, USA) to enrich small RNA fraction. Purified RNA was labelled with Cy3 and hybridization was carried out on miRNA microarray chip (Agilent Technologies, Inc.) containing 15 024 probes, corresponding to 939 human miRNA genes. To exclude extreme outliers, miRNAs with expression more or lower than a threshold (mean fold change >1.2 or <0.8) were eliminated. The remaining data were normalized, mean-centred and log_2_ transformed. After SAM analysis selection, data of 63 miRNAs were assessed using unsupervised hierarchical clustering. Metric (distance) and Linkage were set as ‘euclidean distance’ and ‘Average’, respectively.

### Quantitative reverse transcription and real-time PCR analysis of miRs

Total RNA was reverse transcripted from 50 ng total RNA with MMLV Reverse Transcriptase using following specific primers: RT-miR-195: 5′-GTCGTATCCAGTGCAGGGTCCGAGGTATTCGCACTGGATACGACGCCAAT-3′; RT-miR-497: 5′-GTCGTATCCAGTGCAGGGTCCGAGGTATTCGCACTGGATACGACACAAAC-3′; RT-U6: 5′-CGCT TCACG AATTTGCGTGTCAT-3′. Quantitative reverse transcription and real-time PCR (qRT–PCR) was performed using the ViiA 7 Real-Time PCR System (Applied Biosystems Inc., Foster City, CA, USA) with SYBR Green MasterMix. As a control, the small housekeeping U6 was amplified and quantitated. Gene-specific primers used were miR-195 forward, 5′-GTGCAGGGTCCGAGGT-3′; miR-195 reverse, 5′-GTGCAGGGTCCGAGGT-3′; miR-497 forward, 5′-GTGCAGGGTCCGAGGT-3′; miR-497 reverse, 5′-TAGCCTGCAGCACACTGT GGT-3′; U6 forward, 5′-GCTTCGGCAGCACATATACTAAAAT-3′; U6 reverse, 5′-CGCTTCACGAATTTGCGTGTCAT-3′. Cycle threshold (Ct) values for specific genes were normalized to the Ct value of U6. For miR-15a (Assay ID: 000389), miR-15b (Assay ID: 000390), miR-16 (Assay ID: 000391) and miR-424 (Assay ID: 000604), TaqMan microRNA assay were used according to manufacturer’s instruction (Applied Biosystems).

### qPCR analysis of copy number variations

Genomic DNA was extracted with the Genomic DNA extraction Kit (Macherey-Nagel GmbH, Duren, Germany) according to manufacturer’s instruction. The quality of DNA was assessed by agarose gel electrophoresis. The specific primers used to examine copy number variations of the fragment (78K–15M) of chromosome 17p13.1 were forward, 5′-CCACCTGGCAAGAAGTTTGTG-3′; reverse, 5′-TGTGGTGTTAGAGCGAGGGTG-3′. As a control, the housekeeping HBB was amplified and quantitated. The primers for HBB were forward, 5′-ACACAACTGTGTTCACTAGC-3′; reverse, 5′-CAACTTCATCCACGTTCACC-3′. qPCR was performed using the ViiA 7 Real-Time PCR System (Applied Biosystems) with SYBR Green MasterMix. Cycle threshold (Ct) values were normalized to the Ct value of HGG.

### Array comparative genomic hybridization

One microgram purified high molecular weight DNA from each pair of samples was differentially labelled with Cy3 and Cy5 and co-hybridized to HG18_WG_CGH_v3.1 arrays (Roche Diagnostics, Mannheim, Germany). The array provides measurements from 135 000 unique genomic loci and yields about 22 176 bp of average probe spacing. The aCGH experimental procedure was performed by CapitalBio (Capital-Bio Corp., Beijing, China). aCGH data were extracted and analysed using NimbleScan software and SignalMap software (Roche Diagnostics). To determine the threshold for scoring of gain or loss, normalized, log_2_-transformed ratios were used. Based on the variation in autosomal genomic regions, which should not vary between the two reference samples, thresholds for averaged log_2_ ratio data were set to 0.1 and −0.1 for gains and losses, respectively.

### Plasmid vectors and transfection

The pcDNA-myr-Akt construct, the pBabe-Puro-MEK1-DD (constitutive active MEK1) construct and the pcDNA-IGF1-R construct were purchased from Addgene (Cambridge, MA, USA). Cells were transfected with 2 μg plasmid as well as the empty vector in Opti-MEM medium with Lipofectamine 2000 reagent (Invitrogen, Mulgrave, VIC, Australia) according to manufacturer's protocol.

### Small RNA interference

The small RNA interference (siRNA) constructs for IGF1-R were obtained as the siGENOME SMARTpool reagents siGENOME SMARTpool IGF1-R (M-003012-05-0010; Dharmacon, Lafayette, CO, USA). The non-targeting siRNA control, SiConTRol Non-targeting SiRNA pool (D-001206-13-20) was also obtained from Dharmacon.

### Luciferase reporter assays

IGF1-R-3′UTR and IGF1-R-3′UTR-mut were constructed into pSI-CHECK2-report plasmid (Promega, Madison, WI, USA). Plasmids and miR-195 or miR-497 mimics or anti-miR-195 or anti-miR-497 (Thermo Fisher Scientific) were co-transfected into cells (2.5 × 10^5^) using DharmaFECT Duo Transfection Reagent (Thermo Fisher Scientific). The luciferase activity was measured using the Dual Luciferase Reporter Assay System (Promega) by Synergy 2 multi-detection microplate reader (BioTek, Winooski, VT, USA). Fold-activation values were measured relative to the levels of *Renilla* luciferase activity in cells transfected with negative control oligonucleotides and normalized by luciferase activities.

### Cell viability assays (MTS (3-(4,5-dimethylthiazol-2-yl)-5-(3-carboxymethoxyphenyl)-2-(4-sulfophenyl)-2H-tetrazolium, inner salt) assays)

MTS assays were performed as reported previously.^51^

### Apoptosis

Quantitation of apoptotic cells was carried out by measurement of sub-G1 DNA content using propidium iodide on a flow cytometer as described elsewhere.^52^

### Western blot analysis

Western blot analysis was carried out as described previously.^53^ The intensity of bands was quantitated relative to corresponding GAPDH bands with the Bio-Rad VersaDoc image system (Bio-Rad, Regents Park, NSW, Australia).

### Matrigel invasion assays

Invasion assays were performed by using QCMTM 24-well Cell Invasion Assay Kit with 8 μM membrane (Millipore Merck KGaA, Darmstadt, Germany). Cells were seeded at a density of 1 × 10^5^/chamber well in triplicate and were allowed to invade through the Matrigel for 48 h. Growth medium was applied to both sides. Cells that had invaded to the lower surface of the membrane were dissociated from the membrane, and subsequently lysed and detected by the patented CyQuant GR dye. The fluorescence of invaded cells was quantified by Synergy 2 multi-detection microplate reader (BioTek) using 480/520 nm filter set.

### Statistical analysis

Statistical analysis was carried out using Microsoft Excel 2003 software. Two-tailed Student’s *t*-test was used to assess differences in values between experimental groups. A *P*-value of <0.05 was considered to be statistically significant.

## Supplementary information

Supplementary Information (PDF 897 kb)
