# Supplementary material for: MicroRNA-497 targets insulin-like growth factor 1 receptor and has a tumour suppressive role in human colorectal cancer
Source: Oncogene. 2012 Jun 18;32(15):1910–20. doi: 10.1038/onc.2012.214 (PMC3630484; doi:10.1038/onc.2012.214)
Supplement: Supplementary file 1 — Supplementary Information (PDF 897 kb) [file 41388_2013_BFonc2012214_MOESM46_ESM.pdf]

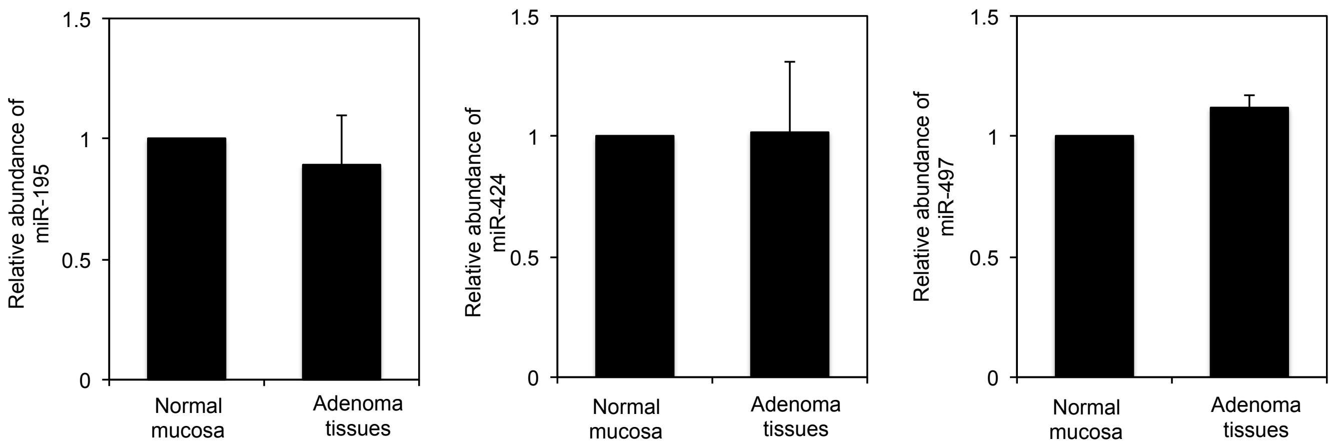

Supplementary Figure 1. qRT-PCR analysis of miR-195 (Left) , miR-424 (Middle), and miR-497 (Right) in total RNA from colon adenoma tissue samples and paired adjacent normal mucosa (n=8) showing that there was no significant difference in the expression of these miRs. The relative abundance of individual miRs in normal mucosa was arbitrarily designated as 1. The data shown are the mean ± SE of three individual experiments.

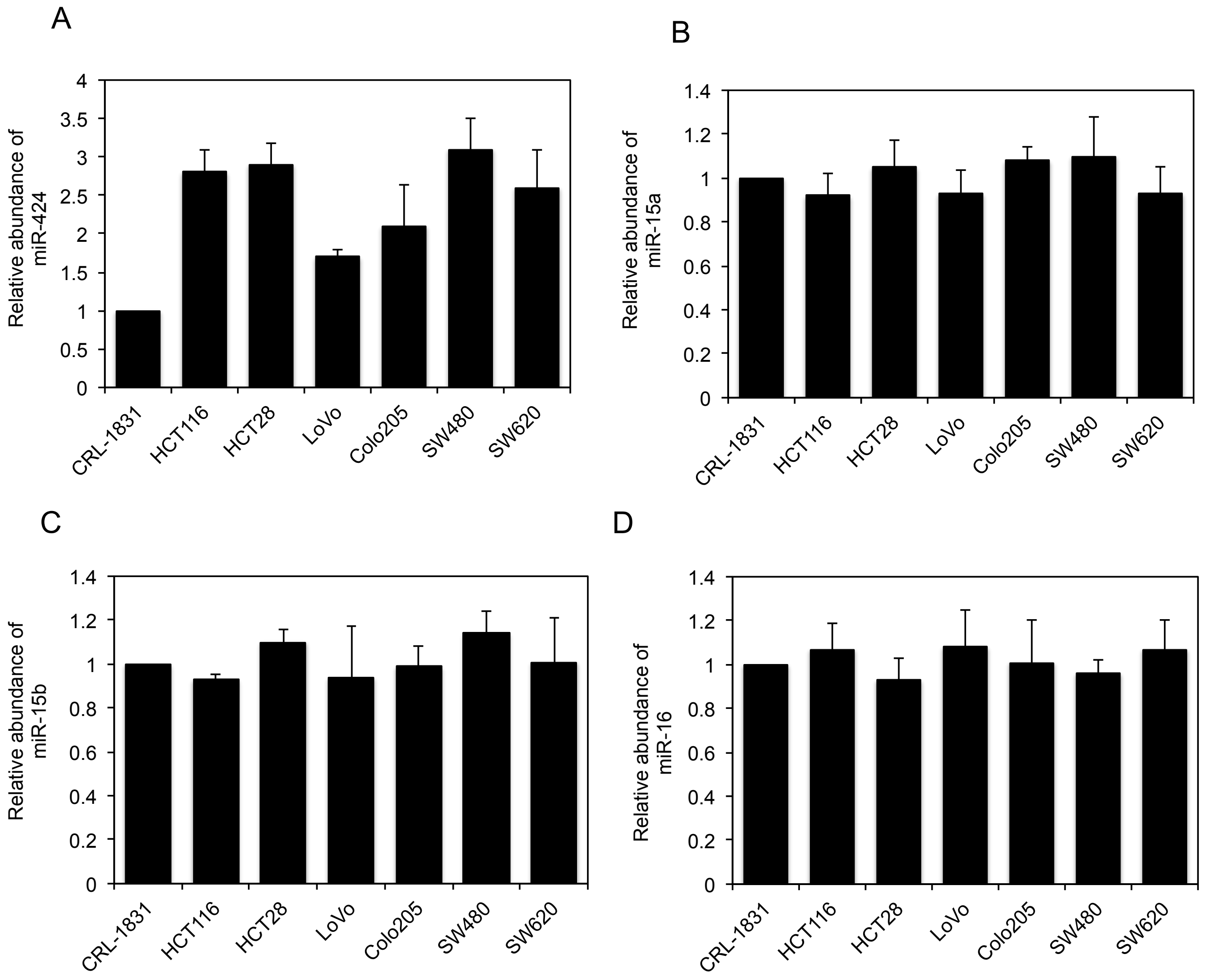

Supplementary Figure 2. qRT-PCR analysis of miR-424 (A) , miR-15a (B), miR-15b (C) and miR-16 (D) in total RNA from indicated colon cancer cell lines and the normal colon epithelial line CRL-1831. The data shown are average fold changes (the mean ± SE of three individual experiments) of individual miR expression in each colon cancer line relative to CRL-1831 cells.

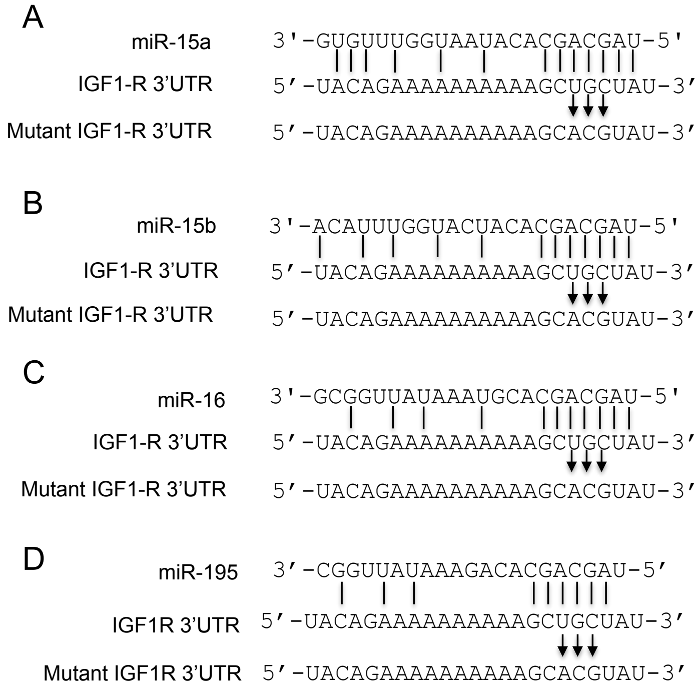

Supplementary Figure 3. A schematic illustration of base-paring between miR-15a (A), miR-15b (B), miR-16 (C), and miR-195 (D) and the 3'UTR of IGF1-R. Substitution of three consecutive bases (UGC to ACG) at the 3'UTR of IGF1-R for the mutant reporter construct is also shown.

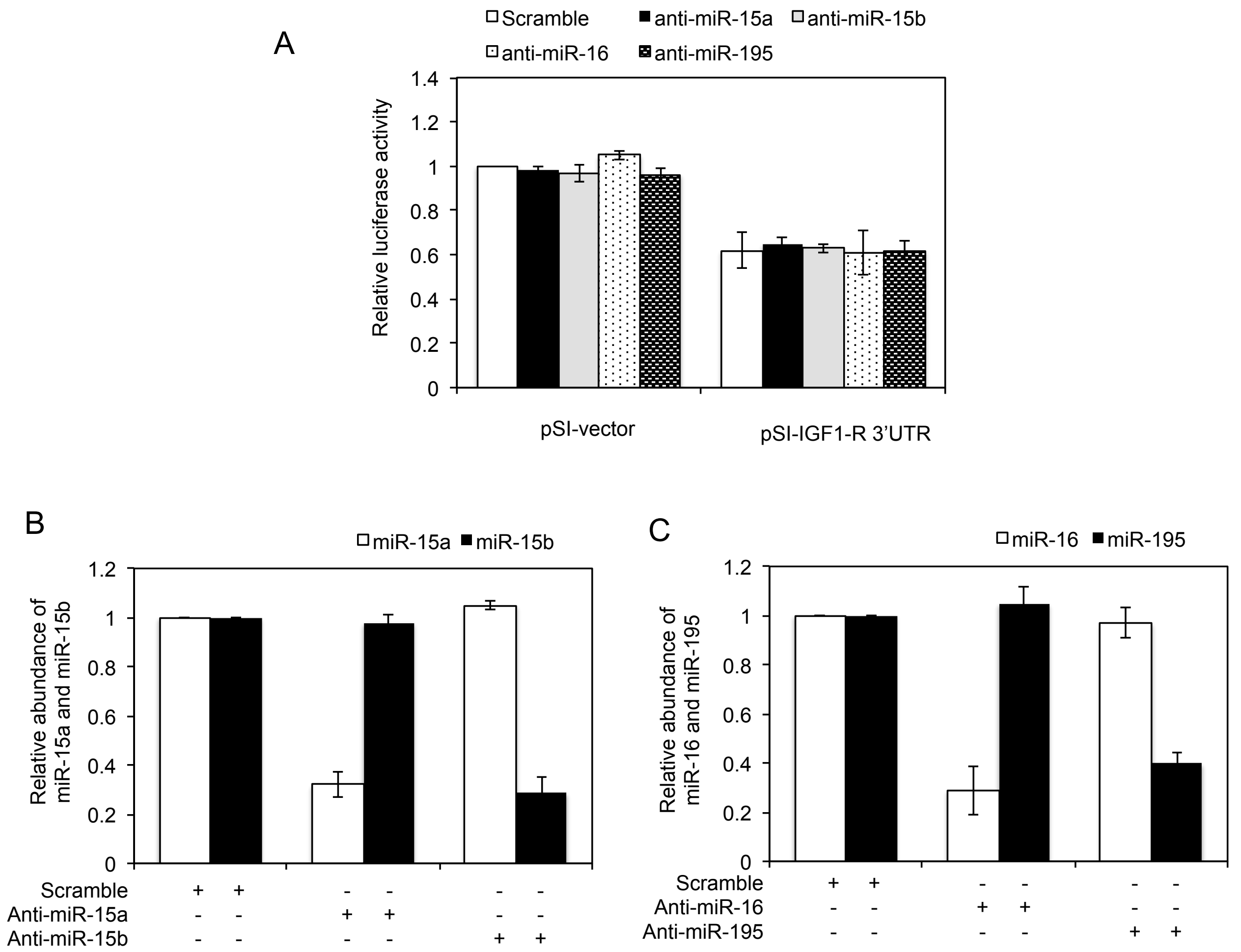

Supplementary Figure 4. A: HCT116 cells were co-transfected with the indicated reporter constructs and renilla luciferase plasmids. Scrambled, anti-miR-15a, anti-miR-15b, anti-miR-16, or anti-miR-195 was also co-transfected. Twenty-four hours later, the reporter activity was measured using luciferase assays. **B:** qRT-PCR analysis of miR-15a and miR-15b in total RNA from HCT116 cells transfected with scrambles, anti-miR-15a, or anti-miR-15b. **C:** qRT-PCR analysis of miR-16 and miR-195 in total RNA from HCT116 cells transfected with scrambles, anti-miR-16 mimics, or anti-miR-195. The data shown are the mean ±SE of three individual experiments.

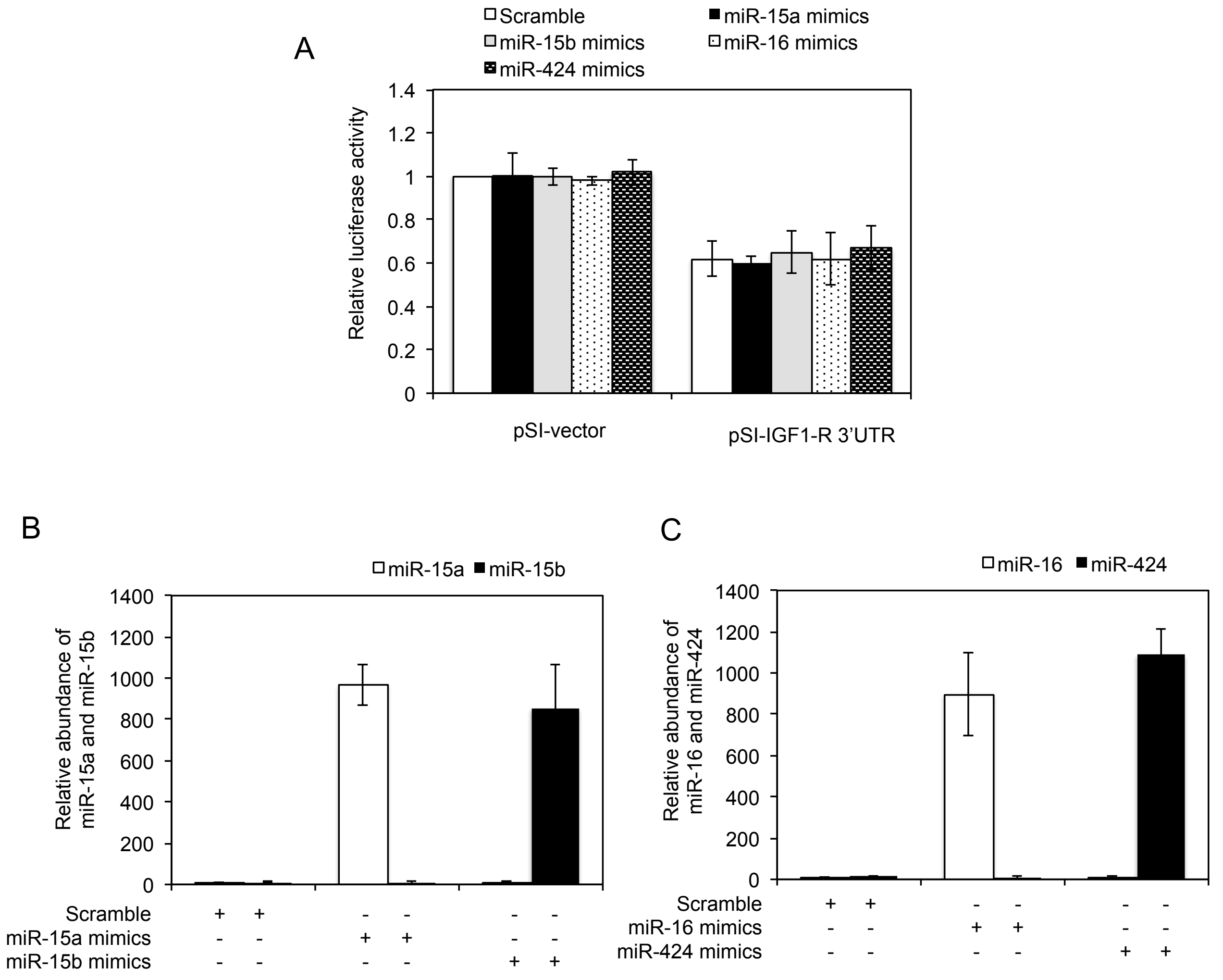

Supplementary Figure 5. A: HCT116 cells were co-transfected with the indicated reporter constructs and renilla luciferase plasmids. Scrambled, miR-15a mimics, miR-15b mimics, miR-16 mimics, or miR-424 mimics was also co-transfected. Twenty-four hours later, the reporter activity was measured using luciferase assays. **B:** qRT-PCR analysis of miR-15a and miR-15b in total RNA from HCT116 cells transfected with scrambles, miR-15a mimics, or miR-15b mimics. . **C:** qRT-PCR analysis of miR-16 and miR-424 in total RNA from HCT116 cells transfected with scrambles, miR-16 mimics, or miR-424 mimics. The data shown are the mean ±SE of three individual experiments.

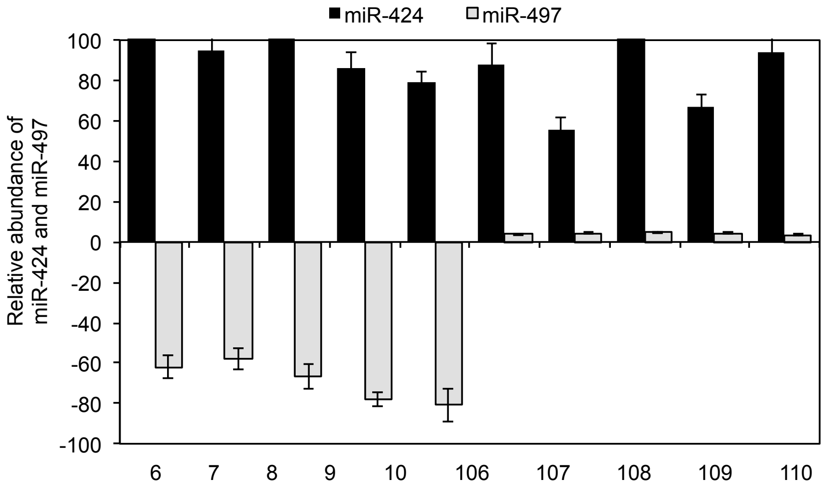

Supplementary Figure 6. qRT-PCR analysis miR-497 and miR-424 in total RNA from CRC tissues and paired adjacent normal colon mucosa sampled from cases listed in Figure 1c. The data shown are average fold changes of individual miR expression in CRC tissues relative to normal mucosa and are the mean ±SE of three individual experiments.

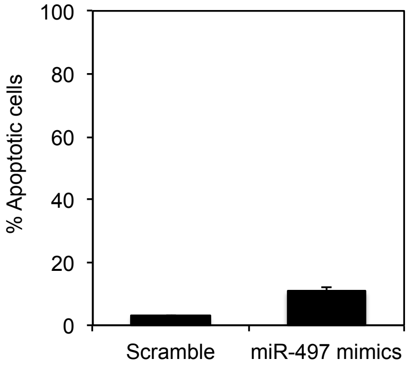

Supplementary Figure 7. Overexpression of miR-497 triggers moderate apoptosis in HCT116 cells. Cells were transfected with scramble sequences or miR-497 mimics. Twenty-four hours later, apoptosis was quantitated by measurement of sub-G1 DNA content with the propidium idido method. The data shown are mean ± SE of three individual experiments.

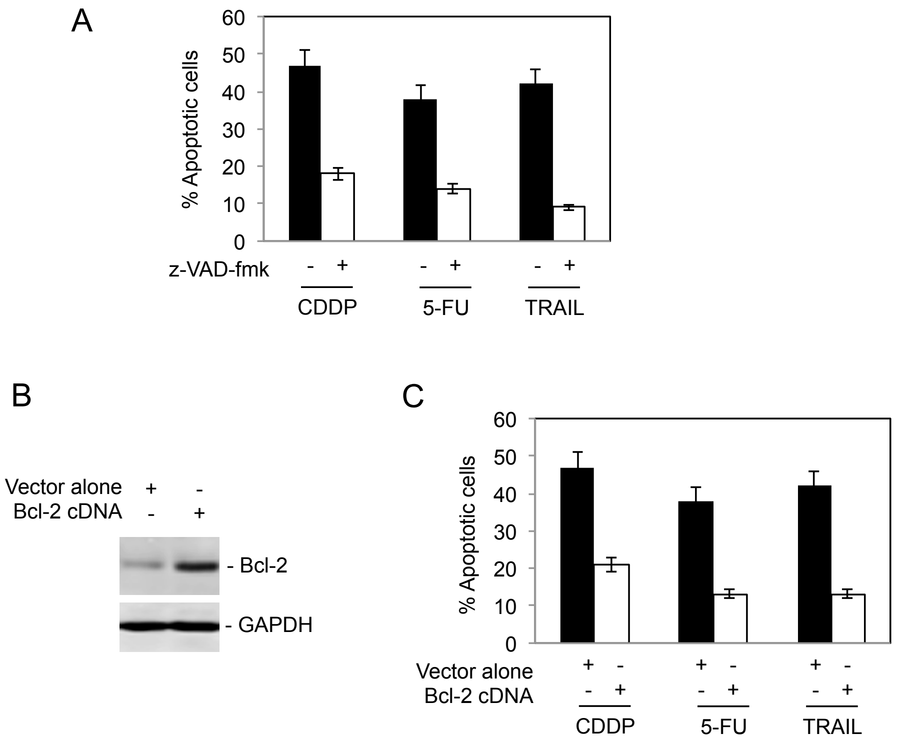

Supplementary Figure 8. A: HCT116 cells were transfected miR-497 mimics. Twenty-four hours later, cells were treated with the general caspase inhibitor z-VAD-fmk (30μM) for 1 hour before with CDDP (5μg/ml) or 5-FU (5mg/ml) for a further 48 hours, or TRAIL (100ng/ml) for a further 24 hours. Apoptosis was quantitated by measurement of sub-G1 DNA content. The data shown are mean ± SEM of 3 individual experiments. **B:** Whole cell lysates from HCT116 cells co-transfected with miR-497 mimics and vector alone or Bcl-2 cDNA were subjected to Western blot analysis of Bcl-2 and GAPDH (as a loading control). The data shown are representative of three individual Western blot analyses. **C:** HCT116 cells were co-transfected with miR-497 mimics and vector alone or Bcl-2 cDNA. Twenty-four hours later, cells were treated with CDDP (5μg/ml) or 5-FU (5mg/ml) for a further 48 hours, or TRAIL (100ng/ml) for a further 24 hours. Apoptosis was quantitated by measurement of sub-G1 DNA content. The data shown are mean ± SEM of 3 individual experiments.

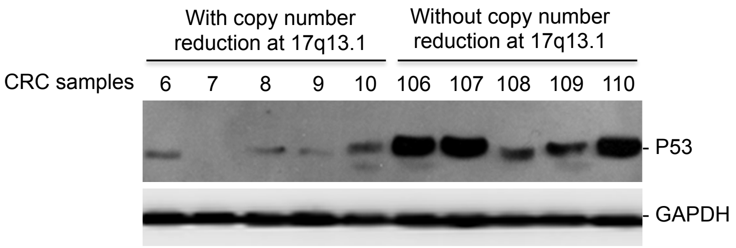

Supplementary Figure 9. Western blot analysis of p53 in whole cell lysates from CRC tissues with or without deletion of the fragment (78K-15M) of chromosome 17p13.1 sampled from CRC tissues shown in Figure 1c. Analysis of GAPDH was included as a loading control. The data shown are representative of three individual Western blots.

Supplementary Table 1, List of miRs that are increased or decreased at least 2-fold in CRC tissues compared to normal mucosa

miRs with increased expression in CRC			miRs with decreased expression in CRC		
miRs	Fold changes	p value*	miRs	Fold changes	p value*
miR-135b	28.987595	9.34E-04	miR-1	19.526276	0.0282
miR-96	8.732975	0.01576417	miR-133b	16.150782	0.018133756
miR-224	7.2610154	0.00255739	miR-145	8.366072	0.024188947
miR-183	6.2726207	0.010806512	miR-133a	7.3642077	0.014471103
miR-645	5.676573	5.67E-05	miR-718	6.76093	0.04126431
miR-592	5.49243	0.022520691	miR-363	6.500951	0.046682566
miR-18a	5.197604	6.92E-04	miR-630	5.5869775	0.04201001
miR-7	4.5956616	0.014782511	miR-1225-5p	5.0466137	0.007815317
miR-18b	4.0509343	8.65E-04	miR-195	4.668555	0.02825547
miR-21	3.8787138	4.88E-04	miR-490-3p	4.0973964	0.035831466
miR-95	3.756966	0.002894311	miR-497	3.7138057	0.0324891
miR-4284	3.0076585	0.00373616	miR-1207-5p	3.5832791	0.012751839
miR-223	2.9948807	0.02141418	miR-338-3p	3.3291068	0.04195197
miR-200b	2.98941	0.04236714	miR-30a	3.1860275	0.032051016
miR-376b	2.9021485	0.004256781	miR-1202	2.749466	0.02706038
miR-362-5p	2.8737812	0.017470311	miR-129-3p	2.5692413	0.030262074
miR-3651	2.613542	8.25E-04	miR-451	2.5443466	0.007334431
miR-20a	2.5521042	0.012881497	miR-378	2.5408177	9.13E-04
miR-34a	2.4578621	0.002889337	miR-29c	2.4427388	0.030578757
miR-425	2.424601	7.04E-04	miR-486-5p	2.4109964	0.004103957
miR-33a	2.4043238	0.00371287	miR-30c	2.2748644	0.028278582
miR-200a	2.403916	0.034788303	miR-4281	2.2080894	0.020005608
miR-429	2.3736513	0.02993104	miR-320c	2.2002711	0.0464227
miR-130b	2.3539615	0.019902224	miR-149	2.1852663	0.02610792
miR-502-5p	2.2805784	0.012245507	miR-3196	2.052178	0.028245434
miR-32	2.2637203	0.02499051			
miR-452	2.2552285	0.007346901			
miR-221	2.2444375	0.003305415			
miR-409-3p	2.2279332	0.00347242			
miR-3654	2.1938884	0.034056693			
miR-210	2.1872356	0.031262822			
miR-146b-5p	2.156597	0.007240667			
miR-20b	2.084619	0.031778198			
miR-93	2.012617	0.020947743			
miR-424	6.580723	0.005074198			
miR-92a	2.0091111	0.008120531			

*p value <0.05 was considered statistically significant.

Supplementary Table 2, Summary of expression of miR-195 and miR-497 in CRC samples of different clinicopathological groups

		Cases	Downregulation of miR-195	p value#	Downregulation of miR-497	p value#	Upregulation of miR-424	p value#
Gender	Male	82	68	0.471	66	0.229	57	0.368
	Female	55	39		40		42	
Age at diagnosis	≤59*	63	49	0.133	46	0.406	39	0.461
	>59	74	58		60		60	
Anatomic location	Ascending colon	25	23	≥0.284	22	≥0.920	19	≥0.615
	Transverse colon	5	5		5		4	
	Descending colon	6	4		4		5	
	Sigmoid colon	24	18		18		16	
	Rectum	77	57		57		55	
TNM stage	I-II	58	49	0.355	46	0.143	43	0.475
	III-IV	79	58		60		56	
Reginal lymph node metastasis	N0	60	49	≥0.318	47	≥0.053	38	≥0.053
	N1	40	34		31		32	
	N2/N3	37	24		28		29	
Distant metastasis	M0	123	95	0.856	94	0.101	88	0.892
	M1	14	12		12		11	

*patients were grouped according to the median age at diagnosis.
